# P-735. Acute Bacterial Skin and Skin Structure Infections: A Comparison of Outpatient One-Hour Infusion Oritavancin and Inpatient Vancomycin Use

**DOI:** 10.1093/ofid/ofae631.931

**Published:** 2025-01-29

**Authors:** William Lindley, Cristen Whittaker, Shana Szymborski, Joseph Reilly, Manish Trivedi

**Affiliations:** AtlantiCare Regional Medical Center, Pomona, New Jersey; AtlantiCare Regional Medical Center, Pomona, New Jersey; AtlantiCare Regional Medical Center, Pomona, New Jersey; AtlantiCare Regional Medical Center, Pomona, New Jersey; AtlantiCare Regional Medical Center, Pomona, New Jersey

## Abstract

**Background:**

Patients with acute bacterial skin and skin structure infections (ABSSSI) who present to the emergency department (ED) are commonly admitted for intravenous (IV) antibiotic treatment. At AtlantiCare Regional Medical Center (ARMC), oritavancin is utilized in the ED as a single 1200 mg dose to prevent ABSSSI admissions. The purpose of this study is to compare 30-day ABSSSI readmission rates between one-hour ED oritavancin infusion and inpatient vancomycin therapy transitioned to oral antibiotics. A financial assessment of both groups will also be presented.

**Table 1.**
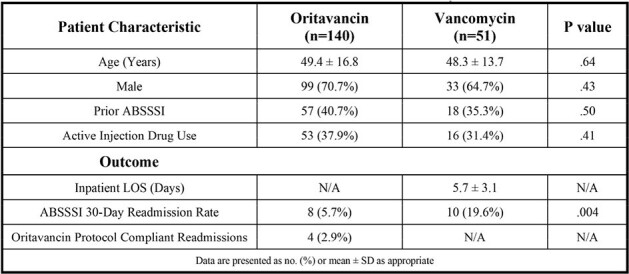
Patient characteristics and outcomes analyzed via Student’s t-test or χ2 test.

**Methods:**

Subjects with ABSSSI were included in this retrospective study if they were administered oritavancin in the ARMC ED between November 2022 and August 2023, or given IV vancomycin as an inpatient between January and August 2023. Data collection included patient demographics, history of ABSSSI, injection drug use, and length of stay (LOS). A financial analysis was performed based on diagnosis-related group (DRG) reimbursement and hospital-specific expenditures. Collected data were analyzed using a Student’s t-test or χ^2^ test as appropriate.

**Results:**

A total of 191 patients were included in this study with 140 and 51 in the oritavancin and vancomycin arms, respectively (Table 1). The 30-day ABSSSI readmission rates were significantly lower in the oritavancin arm (n=8, 5.7%) compared to the vancomycin arm (n=10, 19.6%), p=.004. Amongst the 8 oritavancin readmissions, only 4 (2.9%) were considered oritavancin candidates based on our hospital protocol criteria which defines appropriate patients. The estimated cost after DRG-based reimbursement for a single patient in the vancomycin arm was approximately $10,300. The estimated cost avoidance for each oritavancin patient, not accounting for drug reimbursement or the subsequent decrease in readmissions, was approximately $7,240.

**Conclusion:**

Oritavancin as a one-hour infusion in our ED appears to be an effective treatment option in preventing admissions and significantly decreasing 30-day ABSSSI readmissions. The financial benefits of preventing hospital admissions with single-dose oritavancin should be considered when treating ABSSSI patients in the ED setting.

**Disclosures:**

**Joseph Reilly, B.S., Pharm.D.**, Melinta Therapeutics: Advisor/Consultant

